# Application of crude pectolytic enzymes from *Saccaharomyces cerevisiae* (ATCC 52,712) to starch extraction in Ghana: effects of enzyme technology on pasting characteristics in different cassava varieties

**DOI:** 10.1007/s13197-021-05287-y

**Published:** 2021-11-05

**Authors:** Japheth Kwame Agyepong, John Barimah

**Affiliations:** 1 Department of Biochemistry and Biotechnology, Private Mail Bag (PMB), Kwame Nkrumah University of Science and Technology, Kumasi, Ghana; 2 Department of Food Science and Technology, Private Mail Bag (PMB), Kwame Nkrumah University of Science and Technology, Kumasi, Ghana

**Keywords:** Cassava, Crude pectolytic enzyme, Peak viscosity, Pasting properties, Gelatinization, Polygalacturonase

## Abstract

Previous work on enzyme application to starch extraction enhanced yield and starch recovery rates as well as modified some physicochemical properties of starches for potential alternative application to industry. The response of the technology, however, showed some sensitivity to variety. The knowledge gap therefore was to establish whether such physicochemical responses (by the technology) to variety affects the pasting parameters of the starches extracted. The pasting parameters of starches extracted from four different cassava varieties (‘Nkabom’, ‘Afisiafi’, ‘Bankyehemaa’ and ‘Esambankye’), with the aid of crude pectolytic enzymes from the *Saccharomyces cerevisiae* (ATCC 52,712), were investigated. Although a general response pattern was observed for most of the pasting parameters measured, which includes general enhancements (*P* < 0.05) in starch gelatinization viscosity, with improvements in gelatinization time and temperature and peak viscosities in most of the varieties, there were significant differences (*P* < 0.05) in their respective peak time and temperature requirements for the attainment of peak viscosity. Values for the breakdown viscosity were also generally increased (*P* < 0.05). The technology also increased values for setback viscosity in both the ‘Nkabom’ and ‘Bankyehemaa’ varieties but reduced setback values in the ‘Afisiafi’ and ‘Esambankye’ varieties. As pasting properties are one of the most important characteristics of starch that determine its overall utility, knowledge from this study should inform how adoption of the technology would help diversify the various cassava varieties for appropriate domestic and industrial applications while harnessing its benefits of improved starch yield.

## Introduction

Cassava is one of the most economically important food crops in the world. Together with yam and potato, it is ranked highest in terms of dry matter production per hectare and is considered the ninth most important source of dietary energy (FAO, 2011a). Globally, it is among the six most industrially sought-after agricultural commodity, given global annual production, and the leading contributor of Agricultural Gross Domestic Product (AGDP) in many South America and Sub-Sahara African countries including Ghana (MOFA). Production and processing of the crop to produce starch are fraught with myriads of challenges, especially in Africa where inefficient crude methodologies are employed in starch recovery. It is reported that such traditional extraction methodologies contribute to high post-harvest losses since most of the starch ends up in the root mash residues as agro-waste (Kordylas 1990). In order to mitigate such losses, enzyme-assisted technologies have been adopted to improve recovery rates and yield of starch and such technologies have been reported to be very efficient in many tuber crops (Rahman and Rakshit 2003; Dauito et al. 2005) including cassava (Agyepong and Barimah 2017). Cassava starch has remarkable attributes of forming strong film, clear paste, good water holding properties, and has stable paste viscosity (Nwokocha et al, 2009) which have contributed to its many industrial applications in, for example, the adhesive, pharmaceutical, textile and food industries. In addition, cassava starches do not interfere with flavor compounds in food making them ideal for many food applications. Previous work (Agyepong and Barimah* 2017) to assess effects of the technology on the physicochemical parameters of starches indicated that the enzyme-assisted (starch extraction) technology does not compromise many of the ensuing starches’ physicochemical characteristics and for those physicochemical parameters affected, the technology rather improved them. The technology also proved to be variety-sensitive and, depending on utility, could actually improve starch physicochemical parameters for varied application especially in the food industry. The limitation in our previous reports (Agyepong and Barimah 2017,2018) therefore was to assess the effect of enzyme-based technology on the pasting properties of the starches extracted. Pasting properties of starch determine the stability of the starches during cooking and contribute to starch utility. Regarding pasting parameters, effects of enzyme technology on the many cassava varieties in Ghana is generally poorly understood since in most cases studies in this area is limited to single varieties, usually in isolation. This work therefore seeks to investigate the extent to which enzyme-assisted starch extraction technology affects the pasting qualities of starches extracted from four cassava varieties. We hypothesize that since some physicochemical features of the starches extracted with enzymes were affected and showed also variety-sensitivity in our previous work (Agyepong and Barimah 2018), pasting parameters of starches from the various varieties could likewise be affected. Our main objective, however, is to help inform (given the observed modifications) the choice of cassava variety for enzyme-assisted starch extraction by the local (traditional) extraction industries, with utility of the ensuing starch in mind.

## Methodology

### Plant materials

Root tubers of fresh local cassava (*Manihot esculenta* Cranz) varieties ‘Afisiafi’, ‘Esambankye’, ‘Bankyehemaa’ and ‘Nkabom’ all harvested at nine months after planting (MAP) were obtained under a running project at the Department of Agriculture Engineering, K.N.U.S.T. All varieties were planted on the same field of the Agriculture Research Station (at *Anwomaso-Domeabra*, Kumasi) and had been subjected to similar edaphic and climatic conditions (Agyepong and Barimah 2017).

### Production and assaying of crude pectolytic enzymes

1% Pectic medium was produced using the method described by Ranganna (1986). *S. cerevisiae* (ATCC 52,712) were aseptically cultured in the medium for about 8 days. Over the period, protein content of the medium was monitored using the Biuret test and crude pectolytic enzymes were assayed (Jayani et al. 2005). One unit of polygalacturonase (PGase) activity was as defined by Jayani et al. (2005) as the amount of enzyme required to release 1* μmol* galacturonic acid per minute under standard assay conditions. Pectolytic enzyme activity in the crude enzyme extract was measured at a constant temperature of 28 °C.

### Endogenous amylase enzyme assay

Over the 8-day period, amylase activities in crude enzyme extract was assayed based on modification of the method described by Bernfield (1955).

### Preparation of cassava mash for starch extraction

**Fig. 1:**
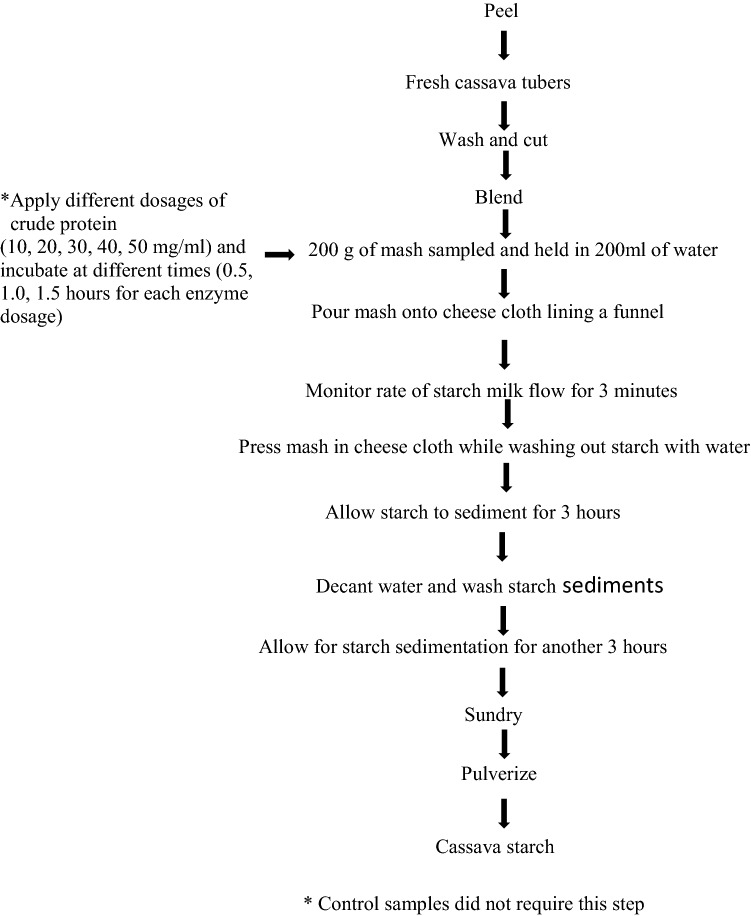
Stages involved in processing cassava root tubers for (enzyme-assisted) starch extraction. (Agyepong and Barimah 2017). Apply different dosages of crude protein (10, 20, 30, 40, 50 mg/ml) and incubate at different times (0.5, 1.0, 1.5 h for each enzyme dosage).* c Control samples did not require this step

Mechanical processes used for enzyme-based starch extraction (for all cassava varieties), as in our previous work (Agyepong and Barimah 2017,2018), is shown in the flow chart (Fig. 1):

Starches obtained from the optimum treatment combinations (retention time vrs crude protein dosage) for yield were sampled and effects of enzyme treatment on their pasting parameters were investigated. Effects of the crude protein extract (enzyme) dosage vrs retention time on yield (Agyepong and Barimah 2017) and physicochemical properties (Agyepong and Barimah 2018) have been reported.

### Determination of pasting characteristics

**Fig. 2:**
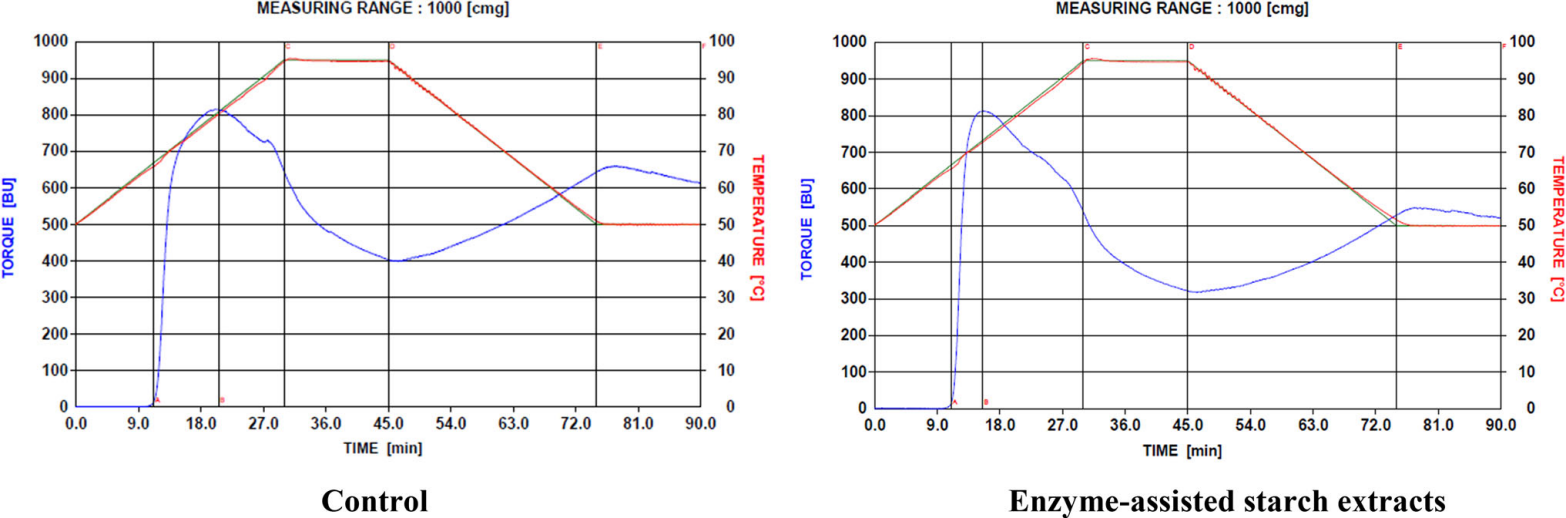
Viscoamylographs of cooked starches extracted from Untreated (Control) and enzyme-treated ‘Afisiafi’ root (pulp) mash

**Fig. 3:**
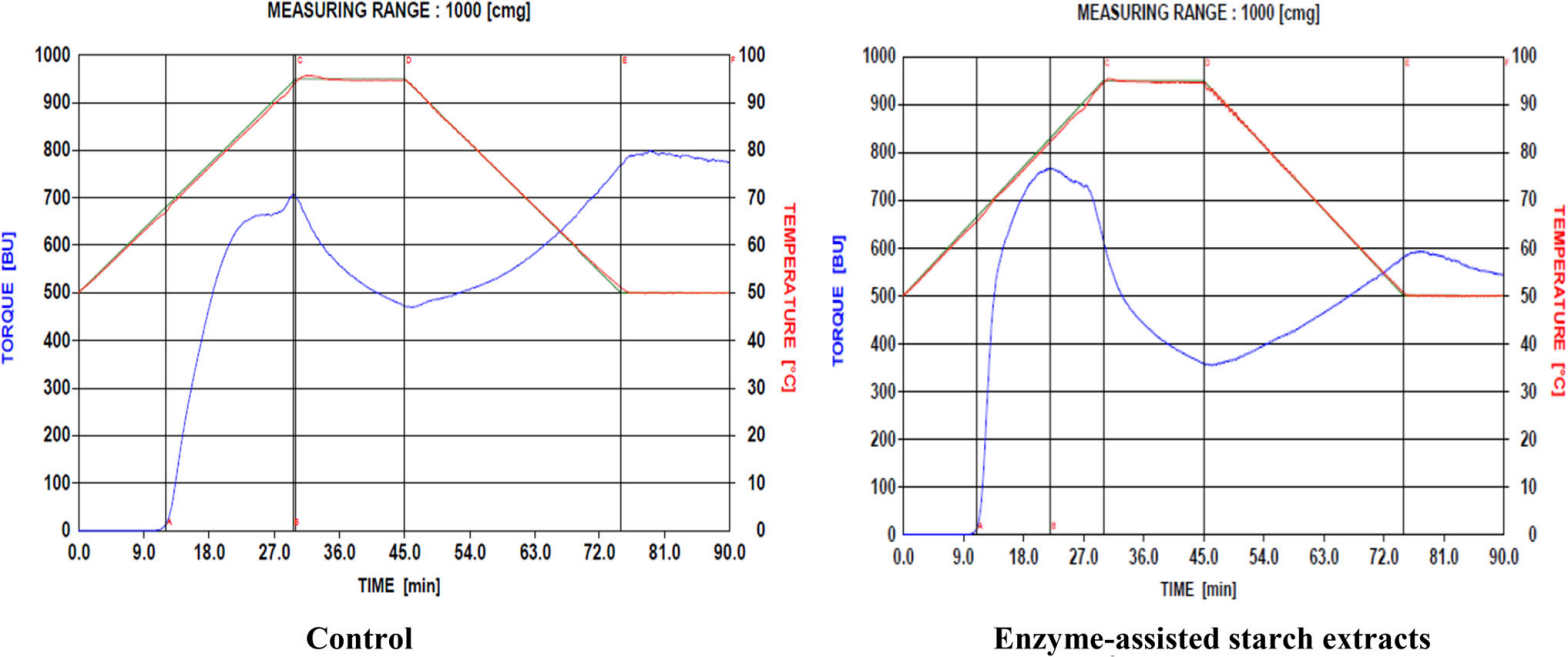
Viscoamylographs of cooked starches extracted from Untreated (Control) and enzyme-treated ‘Esambankye’ root (pulp) mash

**Fig. 4:**
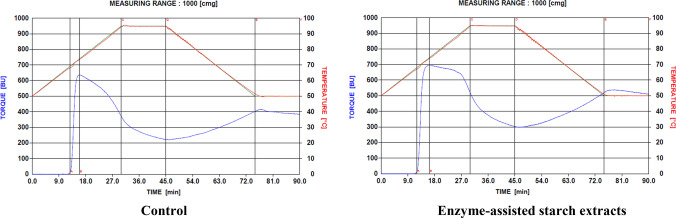
Viscoamylographs of starches extracted from Untreated (Control) and Enzyme-treated ‘Nkabom’ root (pulp) mash

**Fig. 5:**
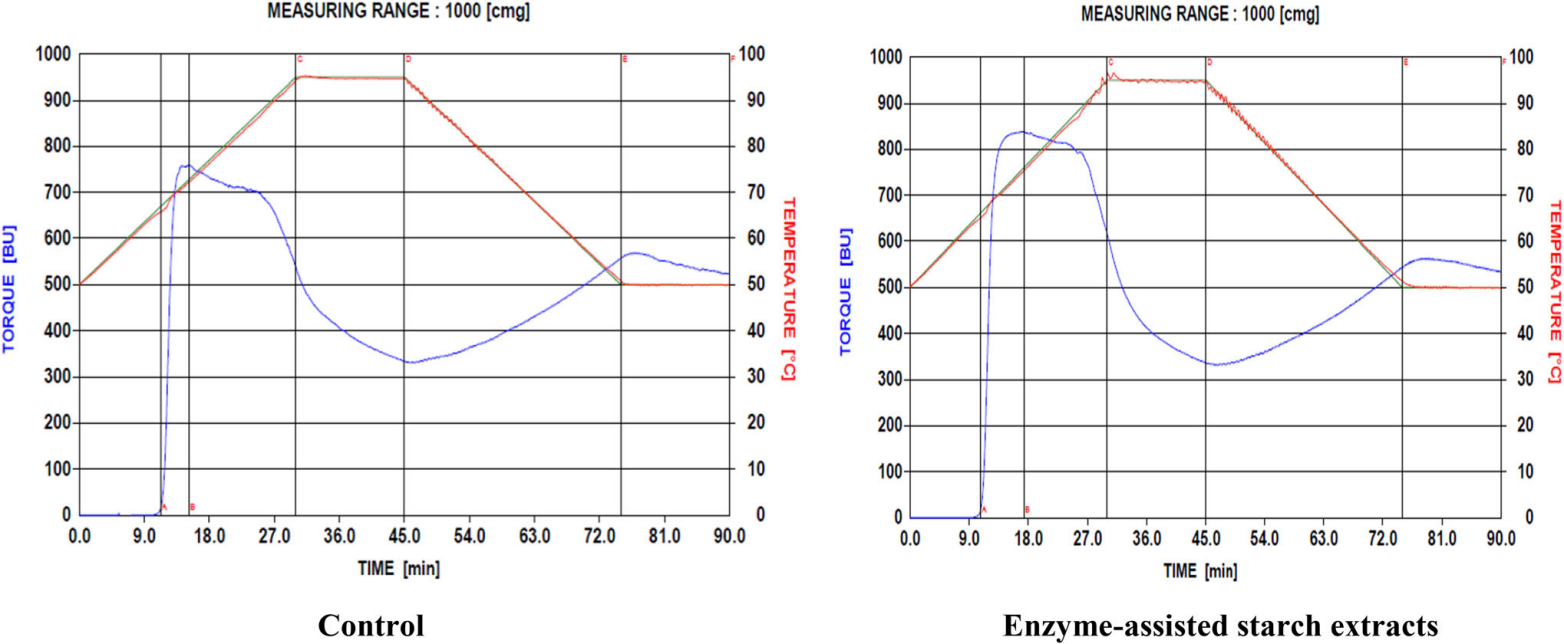
Viscoamylographs of starches extracted from Untreated (Control) and Enzyme-treated ‘Bankyehemaa’ root (pulp) mash

The pasting characteristics of starch samples were determined based on the modification of the Shuey and Tipples (1980) method, with a Brabender visco-amylograph (Brabender OHG Duisburg, Kulturstrabe 51 – 55, D-4100 Duisburg 1) and 700 cmg cartridge. Cassava starch slurry was made by dissolving 30 g of starch (moisture free) in 500 ml of distilled water. The slurry was heated at a rate of 1.5 °C/ min by means of a thermoregulator. When the suspension temperature reached 95 °C, it was held constant for 15 min (first holding period), while being stirred continuously. The paste was then cooled to 50 °C at a rate of 1.5 °C/min and held at this temperature for another 15 min (second holding period). Pasting temperature, peak temperature, peak viscosity, viscosity at 95 °C, viscosity after 15 min at 95 °C, viscosity at 50 °C and viscosity after 15 min at 50 °C were read directly from the viscoamylographs obtained (Figs. 2, 3, 4 and 5). From these, paste stability at 95 °C, paste stability at 50 °C and retrogradation of starches from both enzyme-treated and untreated cassava mashes were determined as:

**Paste Stability at 95**°**C** = viscosity at 95 °C – viscosity at 95 °C (after 15 min)

**Paste Stability at 50**°**C** = viscosity at 50 °C – viscosity at 50 °C (after 15 min)

**Retrogradation (Setback on cooling)** = viscosity at 50 °C –viscosity at 95 °C (after 15 min).

### Statistical tool(s) and analyses

For all parameters measured, statistical analyses were carried out with SigmaPlot for Windows Version 11.0 by Systat Software Inc.^©^ 2008. Completely Randomized Design (CRD) was used to organize all treatments. Data were subjected to a one-way ANOVA (unless otherwise stated) and significant differences were tested using the Duncan’s New Multiple Range Test. Unless otherwise stated, all results were presented as mean ± standard values of three replicates (*n* = 3) and Least Significant Difference (LSD) were also determined for all parameters. Significant differences between treatment means were accepted at *P* < 0.05 (unless otherwise stated).

## Results and discussion

### Enzyme treatment/dosage for cassava mash incubation

After profiling the crude enzyme extracts for optimal starch yield (Agyepong and Barimah 2017), the optimal dosages obtained in Table 1 were applied to the blended mashes from each of the cassava varieties:

**Table 1:** Selected (optimal) enzyme dosages and retention times for cassava mash treatment in the various varieties

Cassava variety	‘Nkabom’	‘Afisiafi’	‘Bankyehemaa’	‘Esambankye’
Enzyme dosage (%)	0.020	0.020	0.025	0.020
Mash retention/Holding time (hours)	0.500	1.000	0.500	0.500

(Agyepong and Barimah 2017)

Pectolytic enzyme activity of 4.91 U and specific activity of 4.291units/mg protein (per minute) were recorded in the crude enzyme extract. However, most of the crude extract’s effects on starch pasting properties were due to the presence of endogenous amylases. An amylase activity of 0.293U, with specific activity of 0.257 units/mg, were obtained (Agyepong and Barimah* 2017) from the crude enzyme extract.

### Pasting properties

Individual viscoamylographs of cooked starches from control and (enzyme) treated root mashes of the various cassava varieties are shown in Figs. 2, 3 and 4 and mean values for these parameters are presented in Table 2:

**Table 2:** Values for pasting parameters of starch samples from enzyme-treated and untreated cassava mashes

Pasting	*Nkabom*		*Afisiafi*		*Bankyehemaa*		*Esambankye*	
**Property**	Control	Treated	Control	Treated	Control	Treated	Control	Treated
Viscosity at gelatinization (BU)	12.5^a^ (3.54)	11.5^a^ (0.71)	13.0^a^ (1.41)	15.0^a^ (4.24)	13.5^a^ (0.71)	15.5^a^ (0.71)	12.5^a^ (0.71)	12.5^a^ (0.71)
Gelatinization temperature (°C)	68.1^a^ (0.14)	66.8^b^ (0.71)	65.8^c^ (0.00)	65.5^d^ (0.14)	65.8^ cd^ (0.71)	65.1^e^ (0.14)	66.9^af^ (0.14)	65.5^d^ (0.71)
Gelatinization time (min)	12.8^a^ (0.12)	11.9^b^ (0.12)	11.25^c^ (0.00)	11.0^d^ (0.06)	11.3^c^ (0.06)	10.8^e^ (0.06)	11.9^b^ (0.12)	11.0^d^ (0.00)
Peak viscosity (BU)	632.5^a^ (6.36)	721.5^b^ (7.48)	815.5^c^ (0.71)	810.5^c^ (4.95)	760.5^d^ (0.71)	838.5^c^ (0.71)	726.0^bd^ (25.46)	768.5^e^ (0.71)
Peak Temperature (°C)	73.4^a^ (0.21)	73.6^a^ (0.28)	80.0^b^ (0.28)	73.2^a^ (0.64)	72.4^d^ (0.21)	75.3^e^ (0.71)	93.7^f^ (0.00)	82.2^ g^ (0.71)
Peak Time (min)	15.8^a^ (0.12)	16.1^a^ (0.24)	20.6^b^ (0.37)	15.8^a^ (0.12)	15.3^a^ (0.24)	17.3^c^ (0.12)	29.6^d^ (0.06)	21.9^e^ (0.12)
Paste Viscosity at 95°c (BU)	375.5^a^ (6.36)	532.5^b^ (6.36)	647.0^c^ (1.41)	537.0^d^ (0.00)	538.5^d^ (0.71)	615.0^e^ (0.00)	719.0^f^ (24.04)	611.0^e^ (4.24)
Paste stability at 95 °C (BU)	150.5^a^ (2.12)	224.5^b^ (12.02)	244.0^c^ (1.41)	218.0^b^ (2.83)	202.5^b^ (3.54)	279.0^f^ (0.00)	245.0^c^ (21.21)	253.0^c^ (4.24)
Viscosity at 50 °C (BU)	407.0^a^ (7.07)	529.0^b^ (12.73)	642.0^c^ (2.83)	516.0^b^ (16.97)	557.5^b^ (0.71)	543.5^b^ (0.71)	770.5^f^ (2.12)	578.0^ g^ (2.82)
Paste stability at 50 °C (BU)	17.5^a^ (0.71)	7.5^b^ (3.54)	32.0^c^ (1.41)	-21.5^d^ (38.89)	31.5^c^ (0.41)	9.0^b^ (1.41)	-4.5^ g^ (2.12)	35.0^c^ (0.00)
Breakdown viscosity	407.5^a^ (10.61)	413.5^a^ (28.99)	412.5^a^ (0.71)	491.5^b^ (14.14)	424.5^a^ (2.12)	502.5^c^ (0.71)	252.0^b^ (22.63)	410.5^a^ (0.71)
Setback viscosity (BU)	182.0^a^ (2.83)	221.0^bh^ (4.24)	239.0^c^ (2.83)	197.0^df^ (14.14)	221.5^eb^ (2.12)	207.5^fh^ (0.71)	296.5^ g^ (0.71)	220.0^eh^ (2.83)

Values in parenthesis () are standard deviations of mean (duplicate) determinations. Means in a row followed by the same letter are not significantly different (*P* > 0.05)

The effects of enzyme treatment on starch stability during processing are key to determining starch utility. Its effects on the behaviour of other pasting parameters such as its viscosity at gelatinization, time and temperature required for maximum viscosity, pasting temperature and peak viscosity are essential. According to Mayiza-Dixon et al. (2005), gelatinization and pasting are the most important properties which influence the quality and aesthetic considerations of starch in the food industry, since they affect texture and digestibility as well as the end use of starchy foods. These parameters must therefore not be compromised by the technology.

#### Effects of enzyme on gelatinization viscosity, pasting time and temperature

Although enzyme treatment did not affect starch viscosity at gelatinization in all varieties (Table 1), the technology decreased the time and temperature required for gel formation. Starch samples from the treated ‘Bankyehemaa’ variety required the least time and temperature for gelatinization (10.8 min and 65.8 °C respectively) while those from the untreated ‘Nkabom’ variety needed the highest (12.8 min and 68.1 °C respectively) (Table 1). Upon investigating the effect of enzyme treatment on granule size, Agyepong and Barimah* (2017) found the technology to enhance the extraction of large size starch granules from the ‘Bankyehemaa’ variety; the ‘Nkabom’ variety was reported among the varieties to release the least size strach granules. Brandam et al. (2003) have reported that starches with larger granule sizes require lower gelatinization temperatures due to the less degree of (amylose) association. Hence the tendency for disruption of molecular interactions between amylose molecules of these starches is high. This enhances their ability to interact with water leading to quicker gelation. Therefore time and energy costs, which are factors critical to profit-making in industrial operations, are both reduced. Thus, enzyme treatment has the effect of reducing gelatinization time and temperature without affecting gelatinization viscosity.

#### Peak viscosity, peak time and temperature

Peak viscosities recorded for all starches were generally higher than reported in most literature (Ikegwu et al. 2009; Nuwamanya et al 2009). The high viscosities might have been influenced by their very low protein content reported earlier (Agyepong and Barimah* 2017). According to Moorthy (2002) high protein content negatively affects pasting properties by causing Maillard reactions which reduce the amount of hydroxyl groups available to interact with water. Starches from enzyme treated varieties had higher peak viscosities than those from their corresponding controls. Peak viscosity of starches from the ‘Afisiafi’ were however not affected by enzyme treatment. It was expected that the release of large size granules by the technology and the inherently high amylose content of starches from the variety should have greatly enhanced peak viscosity of starches from this variety. However, the long incubation time (1.0 h) (Table 1) required for optimal yield (Agyepong and Barimah 2017) could also have compromised starch amylose content (due to long exposure to activities of endogenous amylase in the crude enzyme extract) in the variety. As large starch granules tend to leach out amylose readily at higher temperatures (Lindeboom et al. 2004), it is possible that the longer incubation time allowed for further degradation of pectic substances in the mash, which subsequently culminated in the release of more of the large sized granules (whose amylose interact better with water) to improve viscosity. This also explains the relative ease, in terms of time and temperature requirements, with which starches from the ‘Afisiafi’ attained peak viscosity. For all enzyme-treated samples, starches from the ‘Bankyehemaa’ had the highest peak viscosity while those from the ‘Nkabom’ recorded the least (Table 2). High viscosity is positively correlated with the starch’s inherent amylose-amylopectin ratio and granule size (Kigozi et al. 2013; Lindeboom et al. 2004). Previous reports of ‘Bankyehemaa’ starches having a high amylose content (Agyepong and Barimah* 2017) suggests that amylose from this variety were more resilient to breakdown by the endogenous amylase; its starch granule sizes are also larger compared with those from the other varieties studied (Agyepong and Barimah* 2017).

Peak viscosity measures the point at which gelatinized starches reaches its maximum viscosity before their physical breakdown (Sanni et al. 2004; Tsakama et al. 2010). A desirable property of starch, especially for the food industry, is its ability to attain its maximum viscosity by swelling and subsequently gelatinizing upon cooking. This property is often correlated with final product quality (good texture of paste with moderately high gel strength) (Olanrewaju and Moriyike 2013).

Apart from the ‘Bankyehemaa’ variety, in which the technology increased the time and temperature required for attainment of peak viscosity, starches from the ‘Esam bankye’ and Afisiafi varieties recorded low values for these parameters; the ‘Nkabom’ variety was not significantly affected (*P* > 0.05) (Table 2). Previous study (Agyepong and Barimah* 2017) has suggested that starches from enzyme-treated mashes of the ‘Afisiafi’ and ‘Esambankye’ varieties have relatively high susceptibility to amylose degradation, thus explaining the ease (regarding temperature and time requirements) with which their starches attained peak viscosity; starches from treated root mashes of the ‘Nkabom’ and ‘Bankyehemaa’ are more resilient to amylose breakdown (Agyepong and Barimah* 2017). Although enzyme treatment lowered peak temperature in the ‘Esambankye’ (Table 2), the variety’s starches (both the control and treated mashes) required the highest temperature and time compared with those from the other treated varieties (Table 2).

Regarding the samples' peak viscosity and related time and temperature requirements, enzyme treatment had the most remarkable effect on starches from the ‘Afisiafi’ and ‘Bankyehemaa’ varieties as starches from these varieties recorded the highest peak viscosities (810.5 and 838.5 BU respectively). Starches from the treated ‘Afisiafi’ and ‘Esambankye’ varieties had lower peak temperatures and time requirements; those from the ‘Bankyehemaa’ were higher (*P* > 0.05) and the ‘Nkabom’ was not affected (Table 2). Selection of the ‘Afisiafi’ and ‘Bankyehemaa’ varieties for enzyme-assisted starch production would especially prove beneficial to the adhesive industry where high quality viscose products are desirable and, in terms ofprofit, this will be achieved at lower or similar energy costs. Earlier studies have also reported these varieties as having inherently high starch content and produced higher yields with enzyme treatment (Agyepong and Barimah 2017).

As the technology generally improves the peak viscosities of starches, though at varied temperatures and time, application of the enzyme technology to the food industry clearly has the potential to improve the financial outlook of such industries by enhancing the consistency viscose food products such as marmalades and jams and provide better viscosity profiles to confectionaries that apply glucose, fructose and maltose syrups, gum Arabic, some hard candies and pizza sauce (International Starch Institute, 2020) generally at low energy costs. The technology would also benefit the textile industry in enhancing the quality of fabrics as starch is applied in the sizing, finishing and printing of fabrics. However, the adhesive industry has potential to be most impacted by the technology, especially as it stands to see an improvement in the quality of glue and spray starch products used in binding surfaces.

*Starch Paste viscosity and stability at 95*°*c and Breakdown viscosity*

Individual viscosities of starch samples generally decreased when they were heated to 95 °C. Starches from enzyme-treated ‘Nkabom’ and ‘Bankyehemaa’ varieties (Figs. 4 and 5 respectively) were, however, more viscous than their respective controls (Table 2). The effect of pectolytic enzymes in releasing large starch granules might have contributed to this (Dzogbefia et al. 2008, Agyepong and Barimah* 2017). Viscosity of starches (especially at high temperatures) correlates greatly with starch granule size. At higher temperatures, large-size starch granules disperse and solubilize much easier in water than smaller granules and therefore have a better tendency to associate with water molecules. Their relative resistance to the effects of amylolytic enzymes (Agyepong and Barimah* 2017) might also have contributed to the observed high viscosity. Lindeboom et al. (2004) have reported that both high amylose content and starch granule size correlate positively with viscosity.

Despite reports of pectolytic enzymes enhancing the release of large-size starch granules in the ‘Esambankye’ and ‘Afisiafi’ varieties (Agyepong and Barimah* 2017), their susceptibility to amylolysis (Agyepong and Barimah 2018) might have contributed greatly to the marked reduction in viscosity at 95 °C. The release of large size starch granules (Agyepong and Barimah 2018) possibly rendered most residual amylose more susceptible to further shearing in the shear field of the Brabender (Abera and Rakshit 2003).

After holding the starch pastes at 95 °C, viscosity of starches from all the varieties dropped. A gradual decrease of the paste viscosity during the hold period indicates thermal breakdown of starch and, thus, may be considered a measure of paste stability (Maziya-Dixon et al. 2005). Paste stability explains the resistance of a starch paste to viscosity breakdown as shear is applied at a particular high temperature (usually at 50 °C and 95 °C or above) of the Brabender Visco amylograph. Thus, the differences in viscosity recorded during the holding time were probably due to further granule disintegration and shearing of amylose (subsequently leading to reduction of amylose-water interactions) by the brabender. Except for starch pastes from the treated ‘Afisiafi’ variety (Fig. 2), starches from all other enzyme treated mashes (Figs. 3, 4, and 5) recorded significantly (*P* < 0.05) higher values (Table 2). Higher values for stability at a given holding temperature suggest further granule disintegration and loss of starch crystallinity; it also indicates higher susceptibility to shearing (of amylose) in the shear field of the brabender (Maziya-Dixon et al. 2005). Hence the technology significantly reduced resistance of the starches to disintegration at higher temperatures.

At 95 °C, enzyme treatment least affected starch paste stability in the ‘Esambankye’ variety (Fig. 3 and Table 2); while the stability of starch paste from the treated ‘Bankyehemaa’ was the most impacted, as the variety recorded the highest paste stability value (279 BU) (Table 2). The technology’s effect of enhancing the size of extractable granules (Agyepong and Barimah 2018) could be responsible for this observation as starches with large granule sizes have been reported to easily rupture when cooked to higher temperatures (Maziya-Dixon et al. 2005).

Breakdown values of starches from all the varieties were high (Table 2) suggesting that after attainment of peak viscosity, amylose in all the starches underwent a high degree of fragmentation (Klucinec and Thompson 2002; Adebowale et al. 2005). Breakdown viscosity measures swelling and subsequent disintegration of starches after attainment of peak viscosity; less stable cooked starches usually have high breakdown values (Shimmelis et al. 2006). Thus, the shear stabilities of the starches were therefore generally low. From the initial high peak viscosities observed in the various starches (Table 2), high break down values were expected. Tsakama et al. (2010) have reported a positive correlation between peak viscosity and granule breakdown and also a negative correlation between breakdown viscosity and stability ratio (the ratio between the hot paste viscosity and peak viscosity). In our previous work (Agyepong and Barimah 2018) we observed that enzyme application to starch extraction enhanced the release of starches with large granule sizes. Given the reported ease with which large size granules easily rapture at high temperatures (Maziya-Dixon et al, 2005), the high breakdown values recorded in starches from the treated samples could also be attributed to the effect of the enzyme.

Apart from the ‘Nkabom’ variety that was unaffected, enzyme treatment significantly (*P* < 0.05) increased the breakdown value of starches extracted from all cassava varieties (Table 2) rendering them less viscous. This effect of the enzyme suggests that it would find very good application in the food industry. For instance, the technology could help reduce the viscosity of starches to be used in the preparation and production of custards, gravies and other starch food products that require moderately viscous consistencies (FAO, 2011b) and help improve digestibility. The technology could also be applied to reduce viscosity of starches to be used for low viscosity confectionaries such as gums, jellies and liquorice.

#### Cold paste viscosity (viscosity at 50 °C) and paste stability at 50 °C

Upon cooling to 50 °C (after holding starch samples at 95 °C), there was an increased viscosity in individual starch samples. This was due to the residual amylose undergoing processes that re-associate its moiety to stabilize the paste during the period. Concerning this parameter, starches from the various cassava varieties had different responses: while enzyme treatment reduced the viscosity of starch samples from the ‘Afisiafi’and ‘Esambankye’ varieties (Figs. 2 and 3 respectively), that for the ‘Nkabom’ (Fig. 4) was significantly (*P* < 0.05) increased (Table 2); ‘Bankyehemaa’ starches’ (Fig. 5) viscosity was however not significantly affected (*P* > 0.05) by enzyme treatment (Table 1). The values recorded for viscosity at 50 °C give an indication of the starches’ tendency to retrograde (Safo-Kantanka and Acquistucci 1996; Tipples 1982). Enzyme treatment therefore reduced the retrogradation tendency of starches from the ‘Afisiafi’ and ‘Esambankye’; enhanced retrogradation in the ‘Nkabom’ but did not affect (*P* > 0.05) this parameter in starches from the ‘Bankyehemaa’. Given the varied responses, it can be inferred that response of starches to the technology (in terms of starches’ viscosity at 50 °C) was sensitive to variety.

Except for starch pastes from the ‘Bankyehemaa’ variety that maintained a fairly stable viscosity at 50 °C, significant differences (*P* < 0.05) in the stability of starches from the treated and untreated mashes were recorded (Table 2). Starches from the untreated ‘Esambankye’ and treated ‘Afisiafi’ varieties recorded negative values (Table 2) suggesting that these starches experienced a further increase in viscosity when starches were held at 50 °C temperature. Thus, upon holding the starches at 50 °C, the amylose molecules of the starch pastes from the treated ‘Afisiafi’ and untreated ‘Esam bankye’ mashes quickly re-established strong hydrogen bonding which lead to a further increase in the viscosity of their respective starch pastes at that temperature.

Cold paste viscosity (CPV), or starch viscosity at 50 °C, in starches from the various cassava varieties also gave varied responses to enzyme treatment. Cold paste viscosity is a measure of the viscosity of gelatinized starch after holding it at 50 °C. CPV value was significantly (*P* < 0.05) increased in starches from treated ‘Nkabom’ (Fig. 4) but decreased significantly (*P* < 0.05) in starches from the ‘Afisiafi’ and ‘Esambankye’ varieties (Figs. 2 and 3 respectively); CPV values for starches from the ‘Bankyehemaa’ variety were not affected (*P* > 0.05) by enzyme treatment. For all treated samples, starches from the ‘Afisiafi’, ‘Esambankye’ and ‘Bankyehemaa’ (Figs. 2, 3 and 5 respectively) recorded the highest values for viscosity of the cold paste (CPV) of all treated samples. CPV is important in especially in foods that require cold thickening capacity like instant soups, creams or sauces. High CPV starches are used for instance in the manufacture of noodles (Akindwande et al. 2007). They are also known to affect the textural and sensory properties of food and considered in some food processing operations such as canning (Beta et al. 2001).

### Setback viscosities

The setback viscosity of starches from most untreated varieties recorded significantly (*P* < 0.05) higher values than those from their corresponding treated samples (the only anomaly being starches from the ‘Nkabom’ variety) (Table 2). The small-sized starch granules of the ‘Nkabom’ variety and more importantly their low amylose content as well as their observed resistance to amylase degradation, as reported in our previous work (Agyepong and Barimah 2018), would suggest that their amylopectin moiety was predominant in influencing the starch’s tendency to retrograde. Thus the technology’s relatively low amylolytic effects on the variety (Agyepong and Barimah 2018) resulted in a better realignment of the composite amylose –amylopectin moiety (Zhou et al 2011), eliminating some water from the crystalline structure, further reducing the amylose-water interactions and producing a much ordered structure rather different from its original crystalline form (Wang et al. 2015).

Setback viscosity is a stage where retrogradation or re-ordering of starch molecules occurs after the gelatinized starch has been cooled to 50 °C. Thus, it measures syneresis of starch upon cooling of the cooked starch pastes (Sandhu and Singh 2007). The lower the value, the higher the starch tendency to retrograde (Agunbiade et al. 2011) and form weaker gels. This suggests that the technology reduced the ability of the starch molecules to re-associate and maintain good paste consistency upon cooling. This can be explained in terms of reduction in associative forces (amylose-amylose interaction), by the endogenous enzymes and as a result rather enhancing association of amylose with water. Higher setback values however reduces food digestibility which is desirable and also nutritionally significant, especially for diabetics, as it (further) lowers enzymatic attack on starch helping to release sugar slowly into the blood stream while enhancing its sensory properties (Wang et al. 2015). The values for amylose contents of the starches reported in our previous work (Agyepong and Barimah 2018) generally agree with the setback values recorded (Table 2). Though enzyme application significantly reduced (*P* < 0.05) the setback values in the ‘Bankyehemaa’ variety (Table 2), it was the least impacted (a reduction of 14BU); starches from the ‘Afisiafi’ variety recorded the highest reduction in its setback viscosity (Table 2). Since amylose reassociation at setback depends on the molecular size of amylose (Maziya-Dixon et al. 2005), the relative resilience of ‘Bankyehemaa’ and susceptibility of ‘Afisiafi’ starches to amylolysis, as reported in our previous work (Agyepong and Barimah 2018), would help explain this observation.

## Conclusion

Cooked starches from the various cassava varieties showed different pasting characteristics. Although viscosity at gelatinization was not affected by crude (pectolytic) enzyme application, many other processing parameters (required for cooked starch paste) were impacted differently. Enzyme-assisted extraction generally significantly reduced the time for gelatinization in all starches; those from the enzyme-treated ‘Nkabom’ variety were the most positively impacted as it recorded the highest reduction in its time and temperature for gelatinization. Peak viscosity of starches extracted from the treated root mashes of the ‘Afisiafi’ was not affected; however, those from all other varieties were significantly enhanced. The technology greatly improved the peak viscosity of starches from the ‘Bankyehemaa’ variety at a shorter time but required a higher temperature to attain its peak viscosity. The technology also had varied effects on the viscosities of starches (at 95 °C) obtained from the selected varieties but generally reduced the stability of the viscose starch at 95 °C. Starches from the ‘Bankyehemaa’ and ‘Afisiafi’ varieties recorded the highest paste viscosity and stabilities (at 95 °C) respectively. Effects of enzyme application on starch viscosity and stability at cold temperatures (50 °C) also varied suggesting varietal sensitivity to this parameter. This study has therefore shown that application of enzyme-assisted starch extraction has varied effects on the pasting qualities of starches from different cassava varieties. Overall, the variations observed in the pasting properties of the starches can be attributed to the differences in the biochemical and structural architecture of the starch granules from the different cassava varieties. Application of enzyme technology to starch extraction, however, can help modify the starches pasting properties for various uses. Since industries that depend on starch in their business operations have peculiar viscosity profile requirements, it has become important that such industries closely consider these modifications in pasting parameters to inform their choice of variety. The observed modifications in some pasting parameters therefore suggest that the local starch industry could produce starches tailored for different purposes while benefitting from enhancements in starch yield due to the application of enzyme technology.

## Data Availability

The datasets used and/or analysed during the current study are available from the corresponding author on reasonable request.
